# An Investigation on Forage Yield Capacity of Kermes Oak (*Quercus coccifera* L.) and Grazing Planning of Mediterranean Maquis Scrublands for Traditional Goat Farming

**DOI:** 10.1155/2014/398479

**Published:** 2014-10-14

**Authors:** Ahmet Tolunay, Elif Adıyaman, Ayhan Akyol, Duygu İnce, Türkay Türkoğlu, Veysel Ayhan

**Affiliations:** ^1^Department of Forest Engineering, Faculty of Forestry, Suleyman Demirel University, Eastern Campus, 32260 Isparta, Turkey; ^2^Faculty of Agriculture, Suleyman Demirel University, Eastern Campus, 32260 Isparta, Turkey; ^3^Faculty of Forestry, Suleyman Demirel University, Eastern Campus, 32260 Isparta, Turkey; ^4^Koycegiz Vocational School, Mugla Sıtkı Koçman University, 48800 Mugla, Turkey

## Abstract

This study investigated grazing capacities of maquis scrubland and preparation principles of grazing management in forest resources. Kermes oak (*Quercus coccifera *L.), which is widespread as a main shrub species in maquis vegetation in Turkey, and pure hair goats (*Capra hircus *L.) feeding on shoots and leaves of this shrub were selected for study. The study was conducted in two stages. Green leaf and shoot samples were taken from kermes oaks in the first stage and the amount of green herbage yield (g∗m^−1^) and dry matter yield (kg∗ha^−1^) that may be obtained per unit area from these samples was identified. The considered amount of dry matter consumed by pure hair goats daily and the number of goats being fed within 1 year on land of 1 ha according to different land coverage rates of kermes oaks (goat head∗ha∗yr) were calculated. In the second stage, grazing capacities of sample areas where kermes oak spread were identified and compared with the grazing plan prepared by the forestry administration for this area. Forage yield variance according to land coverage rates of maquis scrublands should be considered when determining optimum animal numbers for grazing per area for sustainable goat farming.

## 1. Introduction

Twenty-seven percent of the overall surface of Turkey is covered with forests and the forest land in the country is 21,537,091 ha. Out of this forest asset, 77% is high forest (16,662,379 ha), while 23% is coppice forest (4,874,712 ha) [1]. Ninety-nine percent of forests are owned by the state and are run and managed by the state [2]. Wood production was adopted as the sole objective in the management of forest resources for the last 50 years and production of nonwood forest products and other services provided by forests were not included. Efforts were made to convert coppice forest into high forest via afforestation activities [3].

Having adopted the principle of multiple uses in the planning of forest resources in recent years, the production of nonwood forest products (water, recreation, wildlife, hunting, etc.) began to be included in the forest management plans in addition to wood production [4]. On the other hand, the products and services provided by coppice forests composed mostly of maquis vegetation were noted as well [5]; maquis vegetation has important ecological functions, such as the prevention of erosion, achievement of hydrological balance, and preservation of biological diversity [6]. Besides the ecological importance of scrublands, they are also of considerable economic importance for supporting livestock by providing nutritious food during critical periods such as summer, when grasses and forbs are dry, and winter, when snow covers the mountain rangelands [7–9].

Goat breeding is one of the traditional occupations, which has been performed for many years in rural regions [10]. The areas in Turkey where pure hair goat breeding is most widely conducted are the Aegean, Mediterranean, and Southeastern Anatolian Regions [11]. Nomads living in these areas have been breeding pure hair goats in the upper basins of that region for centuries [12, 13]. Pure hair goat breeding symbolizes a cultural value for nomads, in addition to being a breeding system [14].

The most prominent plant species spreading within the scrublands in Turkey are as follows: pirnal oak (*Quercus coccifera *L.), boz-pirnal oak (*Quercus aucheri *Jaub. and Spach), broad-leaf jasmine box (*Phillyrea latifolia *L.), terebinth tree (*Pistacia terebinthus *L.), eastern strawberry tree (*Arbutus andrachne *L.), cane apple (*Arbutus unedo *L.), and heather tree (*Erica manipuliflora *Salisb.) [15, 16].

There are similarities between the borders of the regions where pure hair goats are bred and natural distribution borders of some types of trees and shrubs within the Mediterranean scrub vegetation. Shrub vegetation occupies a great part of the territory of the country and the kermes oak (*Quercus coccifera *L.), which is a sclerophyllous shrub, is the dominant species in these scrublands [17, 18].

The forestry policies applied in Turkey aim at reducing and even eliminating breeding pure hair goats on the grounds, which might harm forests [19]. This policy has achieved its purpose up to a certain degree and the number of pure hair goats, which was 15 million in 1975, was reduced to 6 million in 2008 [20].

Over the last few years, however, it has been realized that it is not goats per se that are the real culprit, but the continuous, uncontrolled overgrazing for which humans are responsible [21]. If not managed properly, all domestic animals can damage forests through overgrazing [22].

The Turkish Government enforced an important law in 2011 which eliminated the problems of goat breeding villagers. Radical amendments were made in the relations between goats and forests with this law and villagers were allowed to graze goats in state-owned forests [23]. According to the enforced law, grazing plans should be prepared in order to enable villagers to graze goats in state-owned forests. The law assigned the task of preparing the grazing plans to the forestry administration. The forestry administration prepares the grazing management plans without any scientific basis, considering that 2 goats per 1 ha scrubland will be grazed [24]. Based on some experience obtained from Tunisia and Greece, it is estimated that the grazing capacity is 1.5 goats per 1 ha land [25, 26]. This study was conducted in order to identify the number of animals that may be grazed per unit land in the scrublands in Turkey and develop a method that may be utilized for determining the grazing capacity.

## 2. Materials and Methods

This study was conducted in two stages, namely, the identification of the grazing capacity of kermes oak land and the assessment of the grazing plans prepared by the forestry administration.

### 2.1. Determination of Grazing Capacity of Kermes Oak

#### 2.1.1. Study Area

The first stage of the study was conducted at Süleyman Demirel University, Research and Implementation Forest Areas, in the Province of Isparta, Western Mediterranean Region of Turkey. The study area is located between 37°83′50′′–37°83′31′′ North latitude and 30°51′72′′–30°51′94′′ East longitude and has an elevation of 1,250 m. Its slope is to the southwest. According to data provided by State Meteorology Station of Isparta (SMSI), the long-term average annual rainfall is 600.4 mm and the average air temperature is 12.1°C. During the winter (December–March) and summer (June–September) seasons, the average air temperature ranges from 1.7 to 5.8°C and from 19.7 to 23.1°C and the average rainfall ranges from 90.0 to 100.0 mm and from 9.6 to 36.6 mm, respectively. The climate of the area is characterized by semiarid and cold winters [27]. The soil texture is clay to wet clay, derived from conglomerates of the Mesozoic period and colluvia from river or torrent bank deposits [28]. A range of organic matter content between 2.60 and 3.20% and a pH of 7.5 are both considered average. The shrub variety displaying a native range within the study area is kermes oak. The land coverage rate of kermes oak ranges between 70 and 90% and shrub height ranges between 50 cm and 150 cm.

#### 2.1.2. Experimental Methodology

An experimental area was selected within the university research forest with the same site characteristics (aspect, elevation, slope, soil, etc.). Within this area, kermes oak shrubs being spread over an area of at least 30 m^2^ were identified, and 30 shrubs with this characteristic were selected at random for six periods, namely, April, May, June, July, August, and September. Samples were derived from 30 shrubs in each period (30 × 1 m^2^ = 30 m^2^) and green leaf and shoot samples were obtained from a total land of 180 m^2^ within 6 periods (6 period × 30 m^2^ = 180 m^2^). A sampling quadrant of 1 m × 1 m was created by using wooden slats. Representative, hand-plucked forage samples similar to those consumed by animals were collected [29]. Green herbage samples that had been collected were weighed and, at each period, numerical data was obtained for samples derived from 30 kermes oak shrubs.

In order to determine the dry matter content ratios, 30 herbage samples obtained at each period were mixed into a single sample and were ground in a hammer mill with a sieve hole diameter of 3 mm. In order to determine the dry matter contents, samples were taken from this biomass. All samples were oven-dried at 105°C for 24 hours and weighed [30]. Therefore, the dry matter content of the samples was determined as a percentage ratio. These procedures were conducted in the laboratory separately at each period as 3 parallel and 4 recurrent analyses. The herbage yield obtained from 1 ha was multiplied with the percentage ratios of the dry matter and the dry matter quantity obtained from an area of 1 ha was calculated as kg∗ha^−1^. Thus, the number of animals that may be fed by 1 ha kermes oak was determined upon taking into account the daily dry matter consumption amount of pure hair goats. Moreover, phenological observations were also made on the biological life cycle of kermes oak at this stage.

### 2.2. Evaluation of Grazing Capacity Results on a Grazing Management Plan

The results obtained in the first stage of the study with regard to the optimum number of pure hair goats that may be grazed in kermes oak lands were assessed within the framework of the grazing management plan prepared by the forestry administration for villages where pure hair goat breeding is widely conducted.

#### 2.2.1. Study Area

It is located in the Mediterranean Region of Turkey, within the borders of Isparta Forestry Directorate, Isparta Forestry Operation Directorate, and Central (Isparta) Forestry Sub-District Directorate, and avails the same breeding environment features as with the research area investigated in the first stage. The study area is located between 37°38′33′′–37°03′38′′ North latitude and 30°22′24′′–30°45′34′′ East longitude. The altitude varies between 650 and 2,635 m [31]. The overall area in the grazing management plan prepared by the forestry administration is 98,622.7; 59,008.7 ha of forest land was covered in the management plan while 39,614.0 ha of land (agricultural land, residential areas, and water areas) was excluded from the scope of the plan [32].

The grazing management plan banned animal grazing in 17,233.2 ha forest land while it was allowed in 41,775.5 ha forest land. In the plan, it was assumed that only 2 pure hair goats could be allowed to graze in 1 ha forest land and the number of animals that may be allowed to graze by each village was determined accordingly. The grazing management plan allowed the grazing in forests of 83,459 animals per year in 30 villages [32]. The grazing management plan data prepared by the forestry administration is provided in Table 1.

#### 2.2.2. Experimental Methodology

In areas where pure hair goat grazing is allowed, 10 random trial plots of land of 10 m × 10 m = 100 m^2^ from each individual village were selected. Kermes oak land coverage rate in each plot of land was measured; the average of the measurements was taken to determine the land coverage rate of the trial land. Some parts of the measured fields were those where kermes oaks spread sparsely and which were indicated as forestless soil on the forest management plans. The quantity of these areas was identified and the rate of closure was accepted as 10%. The closure rates (%) obtained as a result of the measurements made in the trial areas (ha) and the size of the fields whose closure is accepted as 10% were taken into account and the kermes oak area coverage rates were identified. These closure rates were used to calculate the optimum number of goats that may be grazed in 1 ha land and optimum number of animals that may be fed in 1 year in the forest lands where grazing is allowed for villagers. The current number of pure hair goats retrieved from the records of Isparta Provincial Agricultural Directorate and the optimum number of animals were compared to identify the villages where there are goats numbers above and below the grazing capacity.

#### 2.2.3. Statistical Analyses

The data relating to the repeated measurements performed in 6 periods for determining the green herbage yield and dry matter yield ratio were subjected to statistical analyses. First of all, it was verified with the Kolmogorov-Smirnov test whether the data were compliant with the regular distribution. It was observed that the distribution of the data relating to all measurements performed in 6 periods according to the results of the Kolmogorov-Smirnov test was compliant with the regular distribution. Therefore, the data were first analyzed upon using repeated ANOVA among parametric methods and the intervals between the periods were identified with the Tukey test. However, as the number [*n*] of samples was 30 in this study and the analysis was performed based on repeated measurements, the Friedman test among the nonparametric tests was conducted. Both parametric and nonparametric analyses provided the same results. [33]. The statistical analyses were carried out using SPSS 16.0 software for Windows [34]. All tests were performed at the level of significance of *P* < 0.05.

## 3. Results

### 3.1. Phenological Observations

The vegetation season began in the middle of March. The buds sprang out and the shoots developed by the end of March. Shoot and leaf development accelerated in April. Blooming began and female flowers were fertilized in May. Leaves which had developed last year began to fall in mid-June. This occurred after the development of the shoots and leaves of the plant and the fertilization of female flowers. Summer drought emerged at the end of June. The top surfaces of leaves became dark green while a wax-like layer formed on the lower surface of leaves.

### 3.2. Green Herbage and Dry Matter Yields

Results of statistical analyses on green herbage yields (in g∗m^−2^ and kg∗ha^−1^), dry matter yield ratio (%), and dry matter yield (kg∗ha^−1^) are presented in Table 2. Differences among the interval means as a result of analyses are statistically significant. Results of the statistical test are shown in Latin letters above the averages.

#### 3.2.1. Green Herbage Yield

The average herbage quantities obtained from an area of 1 m^2^ in different periods were 241.00, 441.83, 574.66, 635.00, 636.33, and 636.66 g∗m^−2^, respectively. Yield of green herbage increased between April and July while no increase occurred in August and September, so it was not found to be meaningful statistically. Furthermore, the green herbage yield values provided as a ratio of g∗m^−2^ in Table 2 were also presented upon being converted into the measurement unit kg∗ha^−1^.

#### 3.2.2. Dry Matter Ratio

Measurements were taken in the periods of April, May, June, July, August, and September; the dry matter ratio of kermes oak was found as 32.03%, 43.26%, 53.83%, 56.85%, 57.35%, and 57.95%, respectively. Dry matter ratio increased between April and July, while no increase occurred in August and September.

#### 3.2.3. Dry Matter Yield

The quantity of dry matter obtained from an area of 1 ha during a vegetation period was 772.0, 1,911.3, 3,093.3, 3,610.0, 3,649.3, and 3,689.4 kg∗ha^−1^, respectively.

### 3.3. Grazing Capacity of Kermes Oak Scrublands

The numbers provided in Table 2 are valid for circumstances when kermes oak land coverage is at 100%. However, under normal circumstances, it may not be possible to find kermes oak scrubland with 100% land coverage and mixed scrub species ratios. Furthermore, it may not be possible to find vegetation where kermes oak is the native variety. Therefore, dry matter yields that may be obtained also for areas where the closure rate varies between 10 and 100% should be calculated. Therefore, Table 3 provides dry matter yields for kermes oak scrubland of 10–100% land coverage.

Using the values of dry matter yields for kermes oak scrubland of 10–100% land coverage, the unit area (1 ha) grazing capacity of kermes oak and the number of pure hair goats that can be grazed in this area were calculated. Pure hair goats consume dry matter that corresponds to 3 to 4% of their live body weight per day. The live body weight of an adult pure hair goat that has grown under Turkey's conditions ranges from 40 to 50 kg. In determining the pure hair goat grazing capacity per unit area of kermes oak scrubland, this study took as a basis a live body weight of 50 kg and estimated that dry matter consumption at 4% of this weight would take place [35]. Accordingly, a pure hair goat weighing 50 kg would consume 2 kg of dry matter per day and 730 kg per year. By dividing the dry matter yields obtained at each period by dry matter consumption of a pure hair goat per year, the number of goats grazing at each period at different land coverage ratios was calculated. The numbers provided in Table 3 were thus obtained.

Accordingly, the number of pure hair goats within an area of 1 ha with a 100% of kermes oak land coverage, which can be fed for 1 year, ranges between 1 and 5. The number of pure hair goats that can be grazed per unit area changes with the ratio of kermes oak land coverage and the quantity of dry matter obtained at different periods.

### 3.4. Selection of the Best Grazing Period

Due to the fact that grazing in areas covered with kermes oaks in countries neighboring the Mediterranean mostly begins and is conducted within the month of June, it seems logical that the figures pertaining to June in Table 3 are used in this study. Using phenological observations, the area researched revealed that the growth roots and development of leaves of the kermes oak take place in April, whereas blooming and fertilization occur in May. As the grazing to be conducted in these periods harms the growth and development of kermes oaks, the yield in herbage and dry matter remain low. In the periods following May, the increase in green herbage stops, the leaves harden due to summer drought, and the shoots become wooden. Thus, June is the most suitable month for grazing in scrublands in pure hair goat breeding [36, 37]. The figures relating to the month of June were used for determining the number of animals that may be allowed to graze per unit area in the next stage of this study.

### 3.5. Results from the Evaluation of a Grazing Management Plan

The coverage rate of kermes oak area on the villages included in the scope of the research within the overall land varies from 10 to 70%. The optimum number of animals in terms of pure hair goats that may be grazed per village is provided in Table 4 upon taking into account the land coverage ratio of maquis species. Hence, the number of goats that may be grazed on an area of 41,775.5 ha was identified as 100,730 goats, whereas this number was identified as 83,549 goats in the grazing management plan prepared by the forestry administration. This result was 17,181 goats less than the optimum number identified in this study.

Considering the current number of 55,672 goats in the villages, it is observed that the grazing capacity is underutilized in 27 villages, while there is unutilized potential that may feed 50,783 goats. Yet, the forestry administration determined the number of villages below capacity as 22 and thus they seem to have the potential to graze only 36,721 goats. These results demonstrate that the forestry administration understated the grazing capacity of kermes oak areas.

The results of the study demonstrate that the number of goats in 3 villages, namely, Buyukhacilar, Harmanoren, and Kucukhacilar, is above the optimum number (^+^5,725 goats) and that overgrazing is done. Whereas, according to the grazing plan prepared by the forestry administration, the number of goats is high (^+^8,844 goats) in 8 villages, namely, Buyukgokceli, Buyukhacilar, Buyukkisla, Guneyce, Harmanoren, Igdecik, Kucukkisla, and Kucukhacilar, this study shows that 5 villages (Buyukgokceli, Buyukkisla, Guneyce, Igdecik, and Kucukkisla) demonstrated to do overgrazing according to the grazing plan are utilized below the optimum capacity.

Breeders should use the grazing areas of neighboring villages where the grazing capacity is underutilized in order to solve the problem of excess goat population (^+^5,725) in villages where grazing is above the optimum capacity. Thus, the excess in the village of Kucukhacilar (^+^3,390 goats) should be shifted to the village of Kuleonu (^−^3439 goats), the excess in the village of Harmanonu (^+^613) should be shifted to the village of Pembeli (^−^916 goats), and the excess in the village of Buyukhacilar (^+^1,782) should be shifted to the grazing field in the village of Sav (^−^2,711 goats).

Pure hair goat breeding should be supported in 27 villages, starting with the villages of Alikoy, Atabey, Darioren, Gonen, Kadilar, and Kucukgokceli where the utilization is close to the optimum capacity or below.

## 4. Discussion

Kermes oak (*Quercus coccifera *L.) was selected in this study as the main species spread in the scrublands in Turkey for the identification of the grazing capacity of scrublands and the assessment of grazing management plans. Calculations were made based on the dry matter yields and closure rates of the kermes oak in the study. In some studies, the dry matter yield rate of the kermes oak for June was found as 51% in the study conducted by Parlak et al. (2011) [38] and Yolcu et al. (2012) [39] and is close to the dry matter rate value obtained in this study.

As mentioned previously, there are also forest areas where kermes oak is mixed with other maquis species. We may question how the grazing capacities of this type of scrubland will be determined and how the grazing plans will be arranged. In the studies conducted recently in the Mediterranean Region, dry matter yields for maquis species where kermes oaks are mixed were calculated.

For instance, the dry matter rate in terebinth trees (*Pistacia terebinthus *L.) was 51.70%, while it was 55.10% in gall oaks (*Quercus infectoria *Oliv.) and 53.11% in broad-leaf jasmine boxes (*Phillyrea latifolia *L.) [39]. As it becomes evident, the dry matter yield rates in the other maquis species are close to the value of kermes oak. Thus, the dry matter yield ratios of kermes oaks may be used for other species in the scrublands. The problem in identifying the grazing capacity in such type of areas is how to determine the green leaf and shoot yields in other species mixed with kermes oak. Samples of green leaves and shoots may be obtained from the areas where such species are spread so as to identify the dry matter yields, as presented in this study. Dry matter yields to be obtained from any area may be calculated upon the determination of the closure or mixture rates.

The study acknowledged that one pure goat would eat dry matter corresponding to 4% of its body weight in determining the number of animals to be grazed in the area per unit. In addition to pure hair goats, Angora goats and Honamli goats are also raised in Turkey but at a lower number. The grazing capacities of the scrublands where grazing is conducted with these animal species should be identified upon taking into account the amount of dry matter required to be consumed on a daily basis.

The study was based on the assumption that pure hair goats were fed only in state-owned forests and did not use other forage resources, because the pure hair goat breeders in Turkey do not hold the economic power to purchase industrial forage. The state provides pure hair goat breeders in Turkey with an annual amount of US$10 per animal bred. The breeders mostly use this subsidy in their household cost of living. Breeders, who are economically better off, purchase forage with this subsidy and spend it for feeding the animals only for 2 months per year, that is, before and after birth.

Some breeders within the study area own extra forage resources as they grow various forage plants in their agricultural land. Thus, as the animals are fed better, this increases the meat and milk yield while the grazing pressure on forest resources is reduced.

Therefore, the state should increase the subsidy provided to the breeders in villages where pure hair goats exceed the grazing capacity, and the villagers involved in the agriculture of forage plants should be supported.

## 5. Conclusions

Grazing animals explore different forage resources to satisfy their daily nutrient needs, following specific spatial and temporal patterns throughout the year [40, 41]. Among them, goats are generally more selective than cattle and sheep depending on the array and quality of plants available [42–44]. Goats are well adapted to the consumption of shrubs inhabiting the Mediterranean forest understory [45, 46]. In particular, pure hair goats prefer browsing [47] and grazing on the shoots and leaves of Mediterranean shrubs in Turkey. Therefore, the forage yield capacity of shoots and leaves of kermes oak which is as a main species in the Mediterranean vegetation has an important role in goat breeding in Turkey.

Integration of pure hair goats in forestry production system is inconceivable without applying proper grazing management. However, this is not easy to achieve because of two main difficulties. One is the need to coordinate goat grazing with the forest management plan. One goal of the forest management plan should be to provide forage for goats on a permanent basis. Since forests are suitable for grazing only for a limited number of years within their rotation time and only for a few months each year, the goat management must be adjusted to these limitations. For this reason, forest areas where grazing is allowed by the government should be classified as a separate working section entitled pure hair goat grazing class in the forest management plans, like timber production. The other difficulty in implementing proper grazing management lies with the people who own and handle goats. Experience has shown that no grazing plan will succeed, no matter how technically perfect it is, if goat shepherds do not accept it. Involving local people in grazing management projects is extremely important. Controlling goat numbers is a fundamental requirement of proper grazing management in forests [12].

To ensure that the pure hair goat breeding system is productive, sustainable, and stable in Turkey, the following become necessary: (1) excessive and irregular grazing conducted by breeders should be stopped; (2) grazing management plans should be prepared according to the results of this research; (3) pure hair goat breeders should accept the grazing program; (4) the forestry administration should inspect whether grazing is performed in a sustainable manner [48].

## Figures and Tables

**Table 1:** Land inventory and prohibited-allowed forest lands of grazing management plan.

Number	Villages	Total village land	Unplanned areas∗	Total forest land	Forest land		Allowed number of goats
					Prohibited grazing land	Allowed grazing land	
		ha	ha	ha	ha	ha	Head goat
1	Alikoy	3,038.3	2,059.8	978.5	82.2	896.3	1793
2	Atabey	13,940.0	2,196.7	11,743.3	1,061.2	10,682.1	2,1364
3	Bayat	1,318.1	1,107.2	210.9	0.0	210.9	422
4	Bozanonu	2,543.8	1,887.3	656.5	208.8	447.7	895
5	Buyukgokceli	2,124.6	1,147.2	977.4	48.2	929.2	1,858
6	Buyukhacilar	2,811.9	786.3	2,025.6	119.7	1,905.9	3,811
7	Buyukkısla	2,379.7	730.2	1,649.5	632.5	1,017.0	2,034
8	Cobanisa	5,680.0	1,334.8	4,345.2	1,583.7	2,761.5	5,523
9	Cunur	3,041.4	1,619.5	1,421.9	730.6	691.3	1,383
10	Darioren	5,326.1	514.1	4,812.0	1,163.1	3,648.9	7,298
11	Direkli	2,214.8	1,062.9	1,151.9	474.0	677.9	1,356
12	Gelincik	1,891.2	734.0	1,157.2	924.6	232.6	463
13	Gonen	10,853.8	5,643.5	5,210.3	1,433.0	3,777.3	7,555
14	Guneyce	1,781.7	335.2	1,446.5	534.8	911.7	1,823
15	Harmanoren	2,104.3	1,536.2	568.1	159.3	408.8	818
16	Igdecik	1,860.1	1,040.9	819.2	117.2	702.0	1,404
17	Islamkoy	2,486.3	2,362.3	124.0	0.4	123.6	247
18	Kadilar	1,836.5	491.7	1,344.8	398.4	946.4	1,893
19	Kapicak	1,902.9	557.3	1,345.6	18.7	1,326.9	2,654
20	Kayi	2,607.9	696.3	1,911.6	1,161.0	750.6	1,501
21	Kizilcik	1,893.6	314.5	1,579.1	905.5	673.6	1,347
22	Koctepe	2,941.5	1,519.3	1,422.2	1,187.7	234.5	469
23	Kuleonu	4,760.7	3,222.1	1,538.6	0.0	1,538.6	3,077
24	Kucukkısla	1,540.5	438.4	1,102.1	232.8	869.3	1,739
25	Kucukgokceli	1,418.5	775.7	642.8	17.6	625.2	1,250
26	Kucukhacilar	756.3	243.7	512.6	0.0	512.6	1,025
27	Pembeli	1,300.3	492.4	807.9	137.1	670.8	1,342
28	Sav	5,434.7	1,620.1	3,814.6	1,209.3	2,605.3	5211
29	Senirce	2,836.0	1,418.5	1,417.5	683.1	734.4	1,469
30	Yakaoren	3,997.2	1,725.9	2,271.3	2,008.7	262.6	525

Total		98,622.7	39,614.0	59,008.7	17,233.2	41,775.5	83,549

^*^Agricultural lands, settlement areas, and water surface; ha: hectare.

**Table 2:** Green herbage yield, dry matter ratio, and dry matter yield results.

Period	Green herbage yields g∗m^−2^		Green herbage yield	Dry matter yield ratio %		Dry matter yield
	Min–Max	Mean ± SD	kg∗ha^−1^	Min–Max	Mean ± SD	kg∗ha^−1^
April	80.0–410.0	241.00^d^ ± 98.83	2410.0	31.75–32.38	32.03^d^ ± 0.32	772.0
May	175.0–750.0	441.83^c^ ± 174.53	4418.3	42.55–43.97	43.26^c^ ± 0.71	1,911.3
June	260.0–890.0	574.66^b^ ± 196.84	5746.6	53.07–54.80	53.83^b^ ± 0.88	3,093.3
July	280.0–980.0	635.00^a^ ± 212.40	6350.0	56.26–57.42	56.85^a^ ± 0.58	3,610.0
August	290.0–990.0	636.33^a^ ± 214.82	6363.3	57.13–57.72	57.35^a^ ± 0.32	3,649.3
September	280.0–980.0	636.66^a^ ± 210.45	6366.6	57.79–58.13	57.95^a^ ± 0.17	3,689.4

a, b, c, and d means in the same column followed by the same letters are not significantly different at the 0.05 level.

SD: standard deviation.

**Table 3:** Dry matter yields and optimal goat capacity of kermes oak in land coverage ratios.

Period	Land coverage ratio									
	10%	20%	30%	40%	50%	60%	70%	80%	90%	100%
April (kg∗ha^−1^)	77.2	154.4	231.6	308.8	386.0	463.2	540.4	617.6	694.8	772.0
(goats∗ha∗yr)	**0.1**	**0.2**	**0.3**	**0.4**	**0.5**	**0.6**	**0.7**	**0.8**	**0.9**	**1.0**
May (kg∗ha^−1^)	191.1	382.2	573.3	764.4	955.5	1,146.6	1,337.7	1,5288	1,719.9	1,911.3
(goats∗ha∗yr)	**0.3**	**0.5**	**0.8**	**1.0**	**1.3**	**1.6**	**1.8**	**2.1**	**2.3**	**2.5**
June (kg∗ha^−1^)	309.3	618.6	927.9	1,237.2	1,546.5	1,855.8	2,165.1	2,477.4	2,783.7	3,093.3
(goats∗ha∗yr)	**0.4**	**0.8**	**1.3**	**1.7**	**2.0**	**2.5**	**3.0**	**3.4**	**3.8**	**4.0**
July (kg∗ha^−1^)	361.0	722.0	1,083.0	1,444.0	1,805.0	2,166.0	2,527.0	2,888.0	3,249.0	3,610.0
(goats∗ha∗yr)	**0.5**	**1.0**	**1.5**	**2.0**	**2.5**	**3.0**	**3.5**	**4.0**	**4.5**	**5.0**
August (kg∗ha^−1^)	364.9	729.8	1,094.7	1,459.6	1,824.5	2,189.4	2,554.3	2,919.2	3,284.1	3,649.3
(goats∗ha∗yr)	**0.5**	**1.0**	**1.5**	**2.0**	**2.5**	**3.0**	**3.5**	**4.0**	**4.5**	**5.0**
September (kg∗ha^−1^)	368.9	737.8	1,106.7	1,475.6	1,844.5	2,213.4	2,582.3	2,951.2	3,320.1	3,689.4
(goats∗ha∗yr)	**0.5**	**1.0**	**1.5**	**2.0**	**2.5**	**3.0**	**3.5**	**4.0**	**4.5**	**5.0**

yr: year.

**Table 4:** Optimum number of pure hair goats that may be bred in villages within the study area and deviations from this number.

Number	Villages	Allowed grazing lands	Land coverage ratio	Goat capacity	Optimum goat capacity	Current goat capacity	Above/below optimum capacity		Grazing plan capacity	Above/below grazing plan capacity		Status
		ha	%	Goats∗ha∗yr	Goats	Goats	Below goats	Above goats	Goats	Below goats	Above goats	
1	Alikoy	896,3	70	3,0	2,689	611	−2,078	—	1,793	−1,182	—	−
2	Atabey	10,682.1	50	2,0	21,364	4,781	−16,583	—	21,364	−16,583	—	−
3	Bayat	210.9	10	0,4	84	NA	−84	—	422	−422	—	−
4	Bozanonu	447.7	40	1,7	761	477	−284	—	895	−418	—	−
5	Buyukgokceli	929.2	70	3,0	2,788	2,063	−725	—	1,858	—	+205	±
6	Buyukhacilar	1,905.9	60	2,5	4,765	8,095	—	+3,330	3,811	—	+4,284	+
7	Buyukkisla	1,017.0	70	3,0	3,051	2,632	−419	—	2,034	—	+598	±
8	Cobanisa	2,761.5	70	3,0	8,285	5,090	−3,195	—	5,523	−433	—	±
9	Cunur	691.3	60	2,5	1,728	1,143	−585	—	1,383	−240	—	−
10	Darioren	3,648.9	60	2,5	9,122	2,528	−6,594	—	7,298	−4,770	—	−
11	Direkli	677.9	50	2,0	1,356	1,233	−123	—	1,356	−123	—	−
12	Gelincik	232.6	40	1,7	395	366	−29	—	463	−97	—	−
13	Gonen	3,777.3	50	2,0	7,555	2,121	−5,434	—	7,555	−5,434	—	−
14	Guneyce	911.7	70	3,0	2,735	1,991	−744	—	1,823	—	+168	±
15	Harmanoren	408.8	70	3,0	1,226	1,839	—	+613	818	—	+1,021	+
16	Igdecik	702.0	70	3,0	2,106	1,520	−586	—	1,404	—	+116	±
17	Islamkoy	123.6	10	0,4	50	42	−8	—	247	−205	—	−
18	Kadilar	946.4	70	3,0	2,839	1,654	−1,185	—	1,893	−239	—	−
19	Kapicak	1,326.9	40	1,7	2,256	1,880	−376	—	2,654	−774	—	−
20	Kayi	750.6	50	2,0	1,501	1,165	−336	—	1,501	−336	—	−
21	Kizilcik	673.6	70	3,0	2,021	1,266	−755	—	1,347	−81	—	−
22	Koctepe	234.5	60	2,5	586	391	−195	—	469	−78	—	−
23	Kuleonu	1,538.6	60	2,5	3,847	408	−3,439	—	3,077	−2,669	—	−
24	Kucukkisla	869.3	70	3,0	2,608	2,152	−456	—	1,739	—	+413	±
25	Kucukgokceli	625.2	70	3,0	1,876	177	−1,699	—	1,250	−1,073	—	−
26	Kucukhacilar	512.6	60	2,5	1,282	3,064	—	+1,782	1,025	—	+2,039	+
27	Pembeli	670.8	60	2,5	1,677	761	−916	—	1,342	−581	—	−
28	Sav	2,605.3	70	3,0	7,816	5,105	−2,711	—	5,211	−106	—	−
29	Senirce	734.4	60	2,5	1,836	606	−1,230	—	1,469	−863	—	−
30	Yakaoren	262.6	50	2,0	525	511	−14	—	525	−14	—	−

Total		41,775.5			100,730	55,672	−50,783	+5,725	83,549	−36,721	+8,844	
